# Association between religiosity and depression varies with age and sex among adults in South America: Evidence from the CESCAS I study

**DOI:** 10.1371/journal.pone.0226622

**Published:** 2019-12-16

**Authors:** Marilina Santero, Federico M. Daray, Carolina Prado, Akram Hernández-Vásquez, Vilma Irazola

**Affiliations:** 1 Department of Chronic Diseases, Institute for Clinical Effectiveness and Health Policy (IECS), Buenos Aires, Argentina; 2 University of Buenos Aires, School of Medicine, Institute of Pharmacology, Buenos Aires, Argentina; 3 National Council of Scientific and Technical Research (CONICET), Buenos Aires, Argentina; 4 Universidad San Ignacio de Loyola, Vicerrectorado de Investigación, Centro de Excelencia en Investigaciones Económicas y Sociales en Salud, Lima, Peru; Monash University, AUSTRALIA

## Abstract

Prior studies have suggest that religiosity mitigates symptoms of depression. However, population-based data in South America are limited. This study determines the prevalence of religiosity and explores its association with depression in four cities of the Southern cone of Latin-America. In the CESCAS I study 7524 participants aged between 35 and 74 years old were recruited between 2011 and 2012 from randomly selected samples in 4 cities (Bariloche and Marcos Paz, Argentina; Temuco, Chile; and Pando-Barros Blancos, Uruguay). Religiosity was assessed with a questionnaire from the Hispanic Community Health Study/Study of Latinos. Two dimensions were used: 1) recognition as belonging to a religion; and 2) frequency of participation in religious activities. Depression was measured using the PHQ-9. Prevalence of religiosity was described by sociodemographic characteristics. Association between religiosity and depression was examined through logistic regression models controlling for sex, age and other potential confounders. Weekly religious activities were reported by 32.3% (95% CI: 30.1, 33.6) of participants. Prevalence of major depressive episode (MDE) was 14.6% (95% CI: 13.6, 15.6). After controlling for confounders, older women (≥65 years) who reported religious affiliation had 70% lower likelihood of having MDE (OR: 0.3; 95% CI, 0.1, 0.8). Moreover, in this group, women participating in religious activities more than once per week compared with “never” had 50% lower likelihood of having a MDE (OR: 0.5; 95% CI: 0.3, 0.9). No association between religious activities and depression was found in men. Religiosity is highly prevalent among adults in four cities of South America. Our study found an inverse association between religiosity and depression only in women, stronger in olders. Although longitudinal studies are necessary to determine the true nature of these relationships, religiosity may be a relevant factor that health care providers could take into account when exploring depression in their patients.

## Introduction

Religiosity is an organized system of beliefs, practices, rituals, and symbols designed to facilitate closeness to the sacred or transcendent[1]. It constitutes a multidimensional phenomenon [2] and construct [3]. LatinAmerica (LA) has 626 millions of inhabitants. Even though there are multiple religions in their territory, more than 425 million of latinaomericans are catholics, the main religion professed–nearly 40% of the world’s total Catholic population[4].

Mental well-being is a fundamental component of World Health Organization´s (WHO) definition of health. Good mental health enables people to realize their potential, cope with the normal stresses of life, work productively, and contribute to their communities [5]. The burden of mental disorders continues growing with significant impacts on health and major social, human rights and economic consequences in all countries of the world. Five types of mental disorders appear within the top 20 causes of Global Burden of Disease (GBD), with major depression being the mental disorder associated with the greatest burden [6]. According to WHO over 350 million people have depression worldwide and on average about 1 in 20 people reported having a major depressive episode (MDE) the previous year [7].The prevalence of depression in epidemiological studies varies according to the methodology used to quantify it, the differences are given depending on the tool used for its detection: symptom questionnaires or structured interviews. A systematic review that included studies in 53 countries, found that when the punctual prevalence of depression was evaluated with symptom questionnaires, the value was 12.1% (95% CI: 9.3; 15.7), while when the 12 months prevalence of the different Affective Disorders (AT) with structured interviews the value was 3.7% (95% CI: 2.7; 5.0)[8]. The prevalence of major depressive disorder (MDD) in South America is 4.0% (95% CI, 3.5, 4.7)[8].

Previous studies have shown that religiosity mitigates symptoms of depression[9–13], possibly by providing a social support system and a stable community of likeminded individuals to serve as a coping mechanism, and providing positive outlooks on stressful situations[14]. Regarding possible mechanisms, it is worthy to note that we still do not know the precise mechanisms, and the listed one are some of the main proposed pathways. Also, it has been described that religious belief is related to health and well-being, particularly among older adults[15, 16]. Religiosity may change with age, and in particular, may become more important to people as they grow older, and their minds turn to the contemplation of the hereafter[17]. Furthermore, one study has found that increased religiosity is inversely correlated with depression in older adults when compare to younger adults[18]. However, this study explore only subjetcs up to 67 years, so age differences in the relationship between religious and depression have not been completely examined.

In spite of the historical and cultural importance of religion and spirituality in the Spanish-speaking populations in most LA countries[19], there is a shortage of works that investigate the prevalence of religiosity and its relationship with age and depression[20, 21]. To our knowledge, no population-based data in the Southern Cone of Latin-America have been published.

Based on previous research, we hypothesis that religiosity increase with age and that there is an inverse association between religiosity and depression. In order to test this hypothesis, we examined data from the CESCAS I cohort study. The objective of our study was to determine the prevalence of religiosity in four cities of the Southern cone of LA, and to explore its association with age and depression.

## Materials and methods

### Study setting and participants

Details of the design and sampling methods of the CESCAS I study have been published elsewhere[22]. Briefly, the study used a 4-stage random sample of a general population of 7,524 adults aged 35 to 74 years from 4 midsized cities in Argentina (Bariloche and Marcos Paz), Chile (Temuco), and Uruguay (Pando-Barros Blancos-Colonia Nicolich). Baseline data were used, in a cross-sectional analysis between February 2011 and December 2012. Of the 10,254 individuals randomly selected, 550 were never found at their homes and 1,394 refused to participate. Of those 8,310 who completed the baseline home surveys, 786 did not attend the clinical examination. Thus, the final sample for this analysis includes 7,524 participants (3,165 men and 4,359 women). The overall response rate was 73.4%, and the response rates were similar in men and women and across different locations.

### Measures

During a home survey, information on religiosity, depression and demographic characteristics were obtained using standard questionnaires.

Religiosity was assessed with a questionnaire from the Hispanic Community Health Study/Study of Latinos[23], cross-culturally adapted for use in Argentina, Chile and Uruguay. Different dimensions of religiosity were examined, in accordance with the National Institute of Mental Health Collaborative Psychiatric Epidemiology Surveys (http://www.icpsr.umich.edu/icpsrweb/CPES/). Dimensions used for this analysis were: 1) if an individual is recognized as belonging to a religion; and 2) religious activities refering to both private and organizational religiousness, measure as frequency of attendance to religious services or other types of participation in religious activities. To assess the frequency of attendance to religiousactivities, participants were asked, “*In general*, *how often do you attend the main worship service of your church or otherwise participate in other activities related with religion (such as watching religious services on TV*, *listening to services on the radio*, *participate in Bible study groups*, *etc*.*)*?” Response options included “*nearly every day*”, “*at least once a week*”, “*a few times a year*”, “*less than once a year*”, or “*never*”. For the analysis these 5 categories were transformed into 3: answers "nearly every day" and "at least once a week" were considered as “at least once a week (≥52)”; answer “a few times a year” was considered as “a few times a year(1–51)”; and answer “less than once a year or never” were considered as “never or less than once a year”[14, 24, 25].

Depression was measured using the PHQ-9. The PHQ-9 is a nine-item self-reported scale, developed to diagnose of MDE as well as to assess severity of depressive symptoms during the two weeks prior to data collection in primary care settings and the community[26]. The definition of an MDE according to the PHQ-9 is based on the DSM-IV diagnostic criteria, which considers at least 2 weeks of persistent depressed mood or anhedonia, accompanied by a total of at least 5 or more of the 9 DSM-IV symptoms of major depression during the episode (significant weight change [5%] or change in appetite; change in sleep [insomnia or hypersomnia]; change in activity [psychomotor agitation or retardation]; fatigue or loss of energy; feelings of worthlessness or excessive or inappropriate guilt; diminished ability to think or concentrate or more indecisiveness; and thoughts of death or suicide) [26]. Each question in the PHQ-9 has four response choices: ‘‘*not at all*”, ‘‘*several days*”, ‘‘*more than half the days*,” and ‘‘*nearly every day*”. This instrument has been validated and calibrated in Argentina[27]. Two scoring systems have been proposed for the PHQ-9 [26]. In the present study, the continuous score was employed by adding up the responses to the nine questions. This way of using the PHQ-9 allows for diagnosis and evaluation of severity of depressive symptoms, the score ranging from 0 to 27. The cut-off point of ≥8 used to determine MDE was based on the calibration of this instrument by our group[27].

### Covariables selection

We identified a set of variables as potential confounders based on a previous work of our group[28]: education (categorized as a 3-level variable: primary, secondary, university); marital status (categorized as married and unmarried if answer single, divorced or widow); health status (categorized as fair/poor versus good, very good or excellent was assessed by scores derived from the locally validated version of the SF-12) [29]; cigarette smoking, and alcohol consumption (obtained using standard questionnaires); stressful life events (SLEs) (determined by asking participants whether they had experienced stressful life events in the past year such as marital separation or divorce, loss of job or retirement, loss of crop or business failure, violence, major intra-family conflict, major personal injury or illness, death or major illness of a close family member, death of a spouse, or other major stress) [30, 31]

### Statistical analysis

The analytic sample comprised 7469 study participants, representing 99.3% of those who were enrolled at baseline; 59 participants were excluded from the analysis because of missing data on depression (n = 51), or frequency of religious attendance(n = 4).

Distribution of frequency of religious activities was characterized using descriptive statistics. Chi-squared test was used to analyze categorical measures, and t–test and analysis of variance were used for continuous measures. Weighted prevalence of MDE by measures of religiosity, age and sex was estimated.

A multivariable logistic regression model was developed to estimate the association of religious affiliation with depression, controlling for confounding variables. The model considered depression (yes/no) as the dependent/outcome variable and religious affiliation (with no affiliation as the reference category) as independent variables. A multivariable logistic regression model was developed to estimate the association of frequency of religious activities with depression, controlling for confounding variables. The model considered depression (yes/no) as the dependent/outcome variable and dummy variables for frequency-of-religious activities categories (with never or less than once a year as the reference category) as independent variables. Age and gender interactions with religious affiliation and religious activities were examined, and a test for a linear trend was performed by fitting the logistic model with frequency of religious activities entered as an ordinal variable (linktest). The models included educational level less than high school (yes or no), marital status (unmarried or married), respondent-assessed health status (fair/poor or not), alcohol and tobacco consumption (yes or no).

All the analyses were performed using Stata 14.2 (StataCorp LP, College Station, TX, USA). Estimates included weights that consider the complex and multistage design[22, 32]. Thus, both the descriptive analyzes and the bivariate and multivariable models were performed using the "*svy*" command from Stata for complex designs.

### Ethics statements

The study complies with the Declaration of Helsinki. The study protocol has been approved by IRBs in all participating institutes in Argentina (Comité de Ética de Protocolos de Investigación del Hospital Zonal de Bariloche and Comité de Ética de Protocolos de Investigación Hospital Italiano), Chile (Comité de Ética Científica de la Dirección de Servicio de Salud Araucanía Sur), Uruguay (Facultad de Medicina Udelar), and United States (Tulane University Biomedical Institutional Review Board). Written informed consent has been obtained from all study participants.

## Results

Among the 7469 participants the mean age was 54.8 (SD = 0.13) years, and 57.9% were female. Almost half (45.4%) had completed a primary school equivalent level of education, while over half (68.3%) were married or living as a couple. The majority (92%), 6,859 participants, identified themselves as religiously affiliated.

**Table 1** presents the frequency distribution of religious activities by population characteristics. As can be seen, 7.7% reported no affiliation and 32.3% reported attending at least once a week, which means more than 51 times per year. Women compared to men were more likely to participate in religious activities at least once a week (38.1% versus 25.8%, p <0.001). Frequency of participating in religious activities increased with age (30.9% at least once a week in persons <65 years old versus 41.9% in age group ≥65 years old). Younger compared to older adults were more likely to report “never” participating in religious activities (33.2% vs 26.0% respectively). Regarding educational level and marital status, no differences were observed in the distribution of religious activities.

**Table 1:** Frequency distribution of religious activities by population characteristics.

Population Characteristics		No afilitation	Religious afiliation		
		n = 605	n = 6,859		
			Frequency of activities		
			(No. Services Attended/yr)		
	Overall		Never or less than once a year	A few times a year	At least once a week (≥52)
			n = 2,428	(1–51)	n = 2,148
				n = 2,265	
	n(%)	% (95% CI)	% (95% CI)	% (95% CI)	% (95% CI)
**Overall**	7,469	7.7 (7.0, 8.4)	32.2 (30.1, 33.6)	27.8 (26.6, 29.1)	32.3 (30.1, 33.6)
**Sex**					
Male	2,738 (36.7)	11.8 (10.6, 13.2)	36.5 (34.6, 38.6)	25.9 (24.1, 27.8)	25.8 (23.9, 27.7)
Female	4,104 (54.9)	4.0 (3.4, 4.6)	28.4 (26.8, 30.1)	29.6 (27.8, 31.3)	38.1 (36.2, 40.0)
**Age group**					
<65years	5,862 (78.5)	7.9 (7.2, 8.7)	33.2 (31.2, 34.7)	28.1 (26.7, 29.6)	30.9 (29.4, 32.4)
≥65years	1,494 (20.0)	6.1 (5.0, 7.4)	26.0 (23.7, 28.3)	26.1 (23.8, 28.5)	41.9 (39.2, 44.7)
**Education**					
Primary	3,162 (42.3)	6.2 (5.4, 7.3)	34.3 (32.4, 36.3)	26.8 (25.1, 28.7)	32.6 (30.7, 34.6)
Secondary	2,533 (33.1)	7.1 (6.2, 8.3)	32.4 (30.4, 34.5)	28.3 (26.3, 30.4)	32.1 (30.0, 34.2)
University	1,141 (15.3)	10.5 (8.9, 12.4)	29.1 (26.3, 32.1)	28.4 (25.6, 31.5)	32.0 (29.0, 35.1)
**Marital status**					
Married or convivent	4,687 (62.8)	7.3 (6.6, 8.2)	31.6 (30.1, 33.2)	28.2 (26.7, 29.7)	32.9 (31.3, 34.5)
Unmarried	2,151 (28.8)	8.5 (7.2, 9.9)	33.7 (31.4, 36.1)	27.0 (24.9, 29.3)	30.8 (28.5, 33.2)

All estimations are weighted to account for the complex survey design.

**Table 2** shows measures of depressive symptomatology by religiosity measures. When the entire population was analyzed together, 14.6% of the individuals reported major depressive episode (MDE). Women compared to man were more likely to report MDE (18.8% vs 9.8% respectively, p <0.05). Regarding religiosity measures, significant differences were found only in women, where never attenders have higher prevalence of MDE compared with those who attend religious services at least once a week or more.

**Table 2:** Weighted prevalence of major depressive episode (mde) by religiosity, age and sex.

		Overall	Female			Male		
	n	% (95% CI)	% (95% CI)			% (95% CI)		
			All	<65	≥65	All	<65	≥65
Overall	7,469	14.6 (13.6, 15.6)	18.8 (17.3, 20.4)	19.3 (17.7, 21.1)	15.4 (13.0, 18.2)	9.8 (8.5, 11.3)	10.0 (8.6, 11.5)	9.0 (6.9, 11.5)
**Type**								
No affiliation	610	12.5 (9.8, 15.8)	17.5 (12.7, 23.8)	16.4 (11.4, 32.1)	28.7 (14.4, 49.1)	10.6 (7.5, 14.7)	10.4 (7.1, 15.0)	12.2 (6.2, 22.8)
Religious	6,859	14.8 (13.7, 15.9)	18.8 (17.3, 20.4)	19.5 (17.7, 21.3)	15.1 (12.6, 17.9)	9.8 (8.5, 11.2)	9.9 (8.5, 11.6)	8.6 (6.5, 11.2)
**Frequency**								
Never or less than once a year	2,428	15.6 (13.9, 17.5)	22.3 (19.5, 25.4)	22.5 (19.4, 25.9)	20.7 (15.4, 27.1)	9.9 (7.9, 12.1)	10.1 (8.0, 12.6)	7.7 (4.8, 12.1)
A few times a year	2,265	14.6 (12.7, 16.8)	19.2 (16.3, 22.4)	20.2 (17.1, 23.8)	11.5 (7.7, 16.8)	8.8 (6.6, 11.6)	8.7 (6.3, 11.8)	9.4 (5.7, 15.2)
At least once a week	2,148	13.9 (12.2, 17.8)	15.8 (13.6, 18.2)	16.1 (13.6, 18.9)	14.4 (10.9, 18.7)	10.7 (8.2, 13.9)	11.1 (8.3, 14.8)	8.5 (5.3, 13.4)

All estimations are weighted to account for complex survey design.

Major depressive episode was defined as a scoring ≥8 in PHQ-9

We examined the association between religiosity (independent variable) and depression (dependent variable) stratified by age group and sex (**Table 3**). Among women (≥ 65 years old) reporting religious affiliation was associated with a 70% lower likelihood of having MDE (OR: 0.3; 95% CI, 0.1, 0.8) in comparison with those reporting no religious affiliation. Moreover, frequent attending religious activities compared to never attending was associated with a 50% lower rate of MDE in women ≥65 years old. This association was found in both groups, those who attend a few times a year and those who attend more frequently. In younger women (<65years old) a protective association with MDE was found only in those who attend to religious activities at least once a week or almost every day (OR: 0.7, 95% CI: 0.5, 0.9). In men, MDE was not associated with religiosity at any age group.

**Table 3:** Association between religiosity and Major Depressive Episode by age group.

	Female (n = 4325)		Male (n = 3144)	
	Age Group		Age Group	
	<65	≥65	<65	≥65
Religiosity	OR (95% CI)	OR (95% CI)	OR (95% CI)	OR (95% CI)^a^
**Affiliation**				
No affiliation	Ref.	Ref.	Ref.	Ref.
Religious	1.1 (0.7, 1.7)	**0.3 (0.1, 0.8)**	0.9 (0.6, 1.5)	0.7 (0.3, 1.6)
**Frequency of religious activities**				
Never or less than once a year (0)	Ref.	Ref.	Ref.	Ref.
A few times a year (1–51)	0.9 (0.7, 1.2)	**0.4 (0.2, 0.8)**	0.9 (0.6, 1.4)	1.22 (0.6, 2.6)
At least once a week or almost every day (52)	**0.7 (0.5, 0.9)**	**0.5 (0.3, 0.9)**	1.4 (0.9, 2.2)	1.0 (0.4, 2.4)

All estimations are weighted to account for complex survey design.

OR (Odds Ratio) describes the odds of having a major depressive episode, defined as DSM-IV diagnostic criteria.

All estimations are adjusted for education, marital status, alcohol, tobacco, stressful life events and health status.

For MDE, interaction terms with age were not statistically significant for affiliation or frequency of religious participation (P = > = 0.05)

Statistically significant results (p<0.05) are highlighted in bold type.

## Discussion

The present study aims to explore the relationship between religiosity and depression using a population-based sample in four cities of South America. Religiosity, as indexed by religious activity and religious affiliation, was highly prevalent in our population of adults from cities of Argentina, Chile and Uruguay when compared to other regions of the world[33]. Frequent religious activity was more prevalent in women than men, and in older age groups than in younger ones. Overall, our data showed no association between religious affiliation and frequency of religious activities, with depression. However, in age-stratified analyses, religious attendance is protective against depression among women and the magnitude of this effect is higher in those older than 65 years old.

Worldwide, weekly religious activities varies within each region[34]. The prevalece found in our study, about 30%, is similar to the prevalence found by other authors [34–37]. Countries in sub-Saharan Africa tend to have the world’s highest levels of regular attendance; in the average country in that region, 79% of adults say they attend services weekly. Across Europe, aside from Poland, where 42% of respondents attend weekly, every other European country has rates of attendance at or below 25%. The values found in our study are are within the expected values for the Americas, where weekly activities ranges from 75% in Guatemala to 14% in Uruguay [33–38]. We found similarities with two particular studies, one in the Latino population living in the United States and the other, in Brazil. In the population-based Hispanic Community Health Study/ Study of Latinos, weekly attendance at religious activities was reported by 41.6% of participants[14]. Other cohort study to investigate the incidence and predictors of health outcomes in an elderly population with low socio-economic level in Brazil, The Bambuí Cohort Study, reported a weekly attendance at religious services of 40.7%[39]. Based on these results, religiosity seems to be highly variable, and our study provides very important data since there is almost no information fromthe Southern Cone.

Several longitudinal studies suggested that individuals tend to become more religious with age[40], and this led to the concept of “Age Gap” around the world, which means that young adults tend to be less religious than their elders[33]. The results of our study are in line with this theory, showing a difference of more than ten percent when comparing religious activities between those under 65 with those over 65 years old. Although the age gap in religious commitment is larger in some countries than in others, it is present in many different economic and social contexts and in societies that are, overall, highly religious as well as those that are comparatively more secular[33]. It has been proposed that religiosity increase with age since older individual engage in religious activities because they seek to cope with stress events, such as illness, loss of loved ones and death[41, 42]. There are also reported gender differences in religious activity among different religious groups[43–45]. It has been suggested that women are more religious than men, but this seems to be culture-specific, and contingent on the measurement method used to measure religiosity[43]. In our study this “Gender Gap” was also observed. Some theories explain gender differences in religion, however a definitive, empirically based explanation of why women generally tend to be more religious than men remain elusive[43]. The CESCAS I study is a population-based study from four representative cities in the Southern Cone of Latin America with a higher female proportion in the sample (57.9%). Comparing sample`s sociodemographic profile with data from general population we found that according to the last census data from each country, the proportion of women is higher than men in Argentina (51.1% in 2018), Chile (50.5%) and Uruguay (51.7% in 2018). In the present study, we observed a prevalence of 14.6% of MDE using a self-reported depressive symptoms as the PHQ-9. This value is within the range observe in a systematic review summarizing the epidemiology of major depressive disorder across 53 countries found, using symptom scale instruments, that point prevalence of depression worldwide was 12.1% (9.3; 15.7)[8].

A growing body of empirical evidence suggests that there is an association between high levels of religious involvement and positive mental health outcomes [1, 2, 46], in particular with depression [47–49]. A meta-analysis of 147 studies involving 98,975 participants found that religiosity is modestly but consistently associated with lower levels of depressive symptoms[10]. There may be many pathways from religiousness to reduce vulnerability to depression[10]. One of these possible pathways is the effect of religiousness on substance abuse, it has been demonstrated that drug abuse may be a risk factor for the development of depression and that religiousness, through moral proscription, is associated with lower rates of substance abuse. Other possible explanation is the effect of religious cognition and behaviors that help individuals to cope with stressful life events that are associated with depression.

Finally, a possible explanation for this association is that religiousness provides people opportunities for social support which has been found to protect against depressive symptoms[10]. As Dengah has found in his research in Brazil[50, 51], religion offers a cohesive world view that provides what the medical sociologist Aron Atonovsky referred to as a “sense of coherence”[52]. That is, people with this cultural model of the world see the world as more predictable and life as unfolding in an anticipated and sensible way, which could be a powerful contributor to better mental health. The results of the present population-based study show that after controlling for potential confounding factors, religious affiliation, measure as frequency of religious activities, is associated with a reduce risk of depression. This association was only observed in women older than 65 years. In this specific group, religious affiliation was associated with a 70% lower risk of suffering a Major Depressive Episode when compared with women without religious affiliation. Moreover, in women (≥65 years), participating in religious services more than once per week compared with “never” was associated with an 50% lower likelihood of having a MDE (OR: 0.5; 95% CI: 0.3, 0.9). These results were consistent with previous findings reported by Lerman et al. (2018) [14] in a population based study of Hispanic/Latino adults (N = 16,415) in four US cities, in which they found an inverse association between religious attendance and depression in women older than 65 years with an effect size was similar or somewhat larger (80%) than the one reported in our study.

The strongest point of our study lies in the fact that CESCAS I is the largest study to provide population-based data on the association of religious activities and depression in locally representative samples of cities of South America, and that the study measured key factors using validated instruments. Despite these strengths, some limitations should be considered when interpreting our results. First, in our study, we used a validated questionnaire from the Hispanic Community Health Study/Study of Latinos which measured only one single domain of religiosity, namely service attendance, and this captures only one aspect of religiosity[14]. At least 12 dimensions of religiousness/spirituality have been defined and instruments developed that measure one or multiple dimensions [3, 53–55]. This analysis might have yielded different results had other dimensions of religiousness been used. Organizations (church attendance) often relates to lower depressions, and private religious practices sometimes refer to higher depression and pain, since they are used as coping strategies by depressed people[56]. Modest over-reporting of religious activities is likely [1]; however, the CESCAS I variable should serve well to separate more frequent from less frequent attenders. Another limitation is that in our study we could not differentiate between different religious affiliation, which might not allow to evaluate the differential effect on depression[56–58], added that the conversion of segments of the population from traditional Catholicism to one of several flavors of Pentecostal Protestantism has been, perhaps, the single most remarked upon phenomenon with respect to religion in South America for over two decades[59]. So, further research is needed, including longitudinal studies of religiousness and mental health in our region. Last, these results are representative of the selected cities included in the study, and therefore do not necessarily represent the general adult population of Argentina, Chile and Uruguay.

## Conclusions

Religiosity is highly prevalent in four cities of the Southern cone of Latin America. Our study found an inverse association between religiosity and depression only in women and stronger in older 65 years old. Although longitudinal studies are necessary to determine the true nature of these relationships, religiosity may be a relevant factor that health care providers could take into account when exploring depression in their patients.

## Supporting information

S1 Table Minidataset. Database and Codebook.
(XLSX)
